# A Pilot Study Using Accelerometers to Characterise the Licking Behaviour of Penned Cattle at a Mineral Block Supplement

**DOI:** 10.3390/ani11041153

**Published:** 2021-04-17

**Authors:** Gamaliel Simanungkalit, Jamie Barwick, Frances Cowley, Robin Dobos, Roger Hegarty

**Affiliations:** 1 Ruminant Research Group (RRG), School of Environmental and Rural Science, University of New England, Armidale, NSW 2351, Australia; fcowley@une.edu.au (F.C.); rhegart3@une.edu.au (R.H.); 2 Precision Agriculture Research Group (PARG), School of Science and Technology, University of New England, Armidale, NSW 2351, Australia; jbarwic2@une.edu.au (J.B.); robin.dobos@dpi.nsw.gov.au (R.D.); 3 Livestock Industries Centre, NSW Department of Primary Industries, University of New England, Armidale, NSW 2351, Australia

**Keywords:** accelerometer, beef cattle, behaviour, licking, mineral block supplements

## Abstract

**Simple Summary:**

Quantifying mineral block supplement intake by individual beef cattle is a challenging task but may enable improved efficiency of supplement use particularly in a grazed system. Estimating time spent licking when cattle access the mineral block supplement can be useful for predicting intake on an individual basis. The advancement of sensor technology has facilitated collection of individual data associated with ingestive behaviours such as feeding and licking duration. This experiment was intended to investigate the effectiveness of wearable tri-axial accelerometers fitted on both neck-collar and ear-tag to identify the licking behaviour of beef cattle by distinguishing it from eating, standing and lying behaviours. The capability of tri-axial accelerometers to classify licking behaviour in beef cattle revealed in this study would offer the possibility of measuring time spent licking and further developing a practical method of estimating mineral block supplement intake by individual grazing cattle.

**Abstract:**

Identifying the licking behaviour in beef cattle may provide a means to measure time spent licking for estimating individual block supplement intake. This study aimed to determine the effectiveness of tri-axial accelerometers deployed in a neck-collar and an ear-tag, to characterise the licking behaviour of beef cattle in individual pens. Four, 2-year-old Angus steers weighing 368 ± 9.3 kg (mean ± SD) were used in a 14-day study. Four machine learning (ML) algorithms (decision trees [DT], random forest [RF], support vector machine [SVM] and *k*-nearest neighbour [kNN]) were employed to develop behaviour classification models using three different ethograms: (1) licking vs. eating vs. standing vs. lying; (2) licking vs. eating vs. inactive; and (3) licking vs. non-licking. Activities were video-recorded from 1000 to 1600 h daily when access to supplement was provided. The RF algorithm exhibited a superior performance in all ethograms across the two deployment modes with an overall accuracy ranging from 88% to 98%. The neck-collar accelerometers had a better performance than the ear-tag accelerometers across all ethograms with sensitivity and positive predictive value (PPV) ranging from 95% to 99% and 91% to 96%, respectively. Overall, the tri-axial accelerometer was capable of identifying licking behaviour of beef cattle in a controlled environment. Further research is required to test the model under actual grazing conditions.

## 1. Introduction

The quantification of mineral block supplement intake by individual cattle will be valuable for improving efficiency of supplement use in grazing systems [1]. Exploiting automatic feeders such as GrowSafe^®^ [2] or SmartFeed^®^ [3] and incorporating chemical markers, such as lithium salts [4] or fenbendazole [5] into the mineral block supplements are common techniques used for estimating consumption by individual cattle. However, their use over a long period in a larger herd is considered impractical and technically prohibitive since not every animal has the chance to access to the feeder bin effectively [3] and the necessity for extensive laboratory analysis of the marker [5]. Hence, advancement of simpler more immediate methods of estimating supplement intake are required to assist managers in decision-making in order to improve efficiency of beef cattle production systems.

Wireless technology using animal-borne sensors allows individual animals to be physically monitored in real-time without interfering in their natural behaviour [6,7]. Tri-axial accelerometers have been routinely deployed to automatically record and classify behaviours of domesticated animals based on the acceleration movements over the three perpendicular axes [8,9,10,11]. Recent investigations have reported that tri-axial accelerometers were capable of categorising oral and intake behaviours of ruminants such as suckling [12], ruminating, eating [13], grazing [14], chewing, biting [11], and drinking [15]. Apart from reducing observation time, the capability of accelerometers to discriminate feeding behaviours indicates the potential for developing algorithms to accurately predict feed intake [16]. Greenwood et al. [17] formulated a simple algorithm to predict pasture intake by individual cattle using accelerometers and Williams et al. [15] reported that accelerometers could be used to predict water intake of grazing cattle based on prediction of visiting frequency and duration per visit to the water trough.

Tri-axial accelerometers have often been affixed to the body parts of beef cattle mainly on the ear (ear-tag) [18], neck (collar) [15] and muzzle (halter) [12]. Several machine learning (ML) algorithms have also been applied to analyse the accelerometer data for developing behaviour classification models in cattle such as decision tree [9,13,19], random forest [20,21], kernel support vector machine [22,23], discriminant analysis, and *k*-nearest neighbours [23,24]. These algorithms generated diverse performances of the models depending mainly on the types of behaviour and sensor placement modes [24,25]. By using neck collar-based accelerometers, Williams et al. [26] succeeded in differentiating drinking from standing (100% accuracy) and walking (92% accuracy) events. However, Kour et al. [12] reported that fitting the accelerometer on a neck-collar was ineffective for classifying suckling behaviour in beef calves. Wolfger et al. [18] found that the ear-tag based accelerometers were able to classify feeding behaviour of lot-fed cattle along with ruminating, active, and resting behaviours with 95% sensitivity and 98% negative predictive value.

Providing supplemental feeds for range cattle in the form of lick-block or loose-lick minerals containing urea during the dry season or phosphorus during the wet season is fundamental to successful cattle breeding in the tropical area of northern Australia [27,28]. The effectiveness of strategic supplementation is contingent upon the ability to decrease between- and within-animal (across days) intake variation [1]. Because grazing cattle mostly ingest such supplements through licking [29], identifying and monitoring this behaviour would be useful to determine whether or not individual animals can meet a targeted consumption, or to place an upper limit on access to a supplement. Simanungkalit et al. [5] has previously shown that time spent at mineral blocks measured by an automatic supplement weighing unit was proportional to block intake on a herd basis. However, high deviation obtained from their linear association was found because of exploratory time before licking. Hence, identifying whether or not the animal is licking while visiting the block supplements is pivotal for improved accuracy of intake prediction. The capability of tri-axial accelerometers to classify behaviour in cattle may offer potential to quantify licking events and time spent licking for the prediction of mineral block supplement intake by individual cattle.

To the best of the authors’ knowledge, no studies have been reported to differentiate licking from other behaviours using tri-axial accelerometers in beef cattle. Hence, this pilot study aimed to determine the effectiveness of tri-axial accelerometers deployed on a neck collar and an ear-tag to characterise the licking behaviour of individually penned beef cattle at a mineral block supplement by distinguishing between licking and other observed (eating, standing, and lying) behaviours. To assess the performance of each deployment mode, four ML algorithms were used to develop behaviour classification models using three different sets of ethograms.

## 2. Materials and Methods

### 2.1. Animals and Experimental Site

Research protocols and use of animals were approved by University of New England (UNE) Animal Ethics Committee (AEC19-041) in accordance with the Australian Code for the Care and Use of Animals for Scientific Purposes. The experiment was conducted at UNE, Armidale, NSW, Australia (30°29′02.3″ S, 151°38′18.5″ E). Four Angus steers aged 2 years with an average body weight (± SD) of 368 (±9.3 kg) were subjects for this study. All steers had been retained and grazed together for six months before the experiment.

### 2.2. Instrumentation

**Figure 1:**
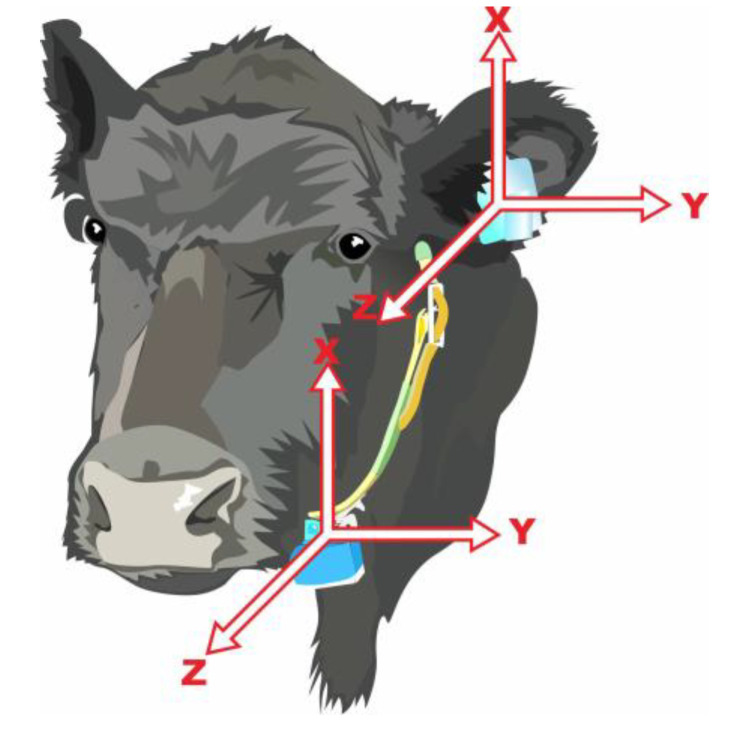
Orientation of the tri-axial accelerometers when attached to both the ear and the neck. Both deployments had the same axis orientation.

Ear-tags and neck-collars equipped with tri-axial accelerometers (AX3 3-Axis Logging Accelerometer, Axivity Ltd., Newcastle Helix, Newcastle, UK) were fitted to all four animals. The ear-tag was attached to the ventral side of the offside left ear and the neck-collar was placed around the neck with the accelerometer mounted on the base of the collar under the lower jaw (Figure 1). Each accelerometer weighed 11 g and has dimensions of 32.5 mm (length) × 23 mm (width) × 7.6 mm (height). The sensors were configured at a sampling rate of 25 Hz (25 records per second) and time-synchronised to a computer clock based on Australian Eastern Daylight Time (AEDT). The expected battery life at this setting was approximately 35 days. Cattle movement was captured through static and dynamic accelerations (gravity; *g*) recorded over the three perpendicular axes of X (vertical; dorso-ventral), Y (horizontal; medio-lateral) and Z (longitudinal; anterior-posterior) (Figure 1). The accelerometer data was temporarily stored on a 512 MB non-volatile flash memory within the sensor in a .cwa file format. At the end of the study, both ear-tags and neck-collars were removed and the accelerometer data were downloaded and converted to a .csv file format using the proprietary software (OmGUI version 1.0.0.43, Axivity Ltd., Newcastle Helix, Newcastle, UK).

### 2.3. Experimental Procedures and Observations

**Figure 2:**
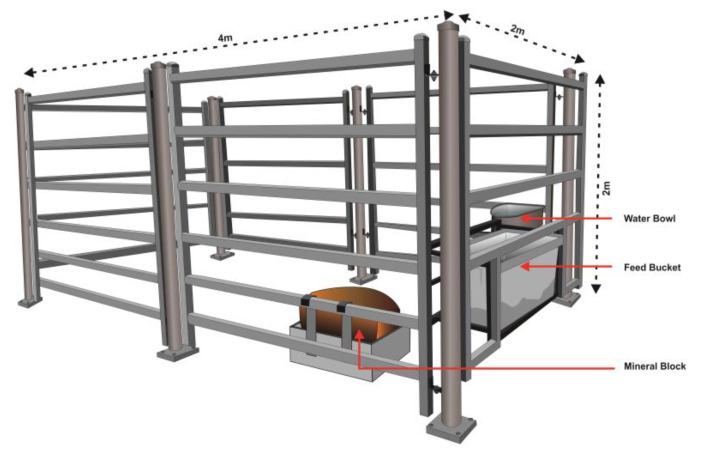
The layout of the individual pen where each animal was confined during the experimental period with a mineral block supplement restrictively provided.

This study was conducted over 14 days. The first seven days involved a habituation period then followed by a seven-day experimental period. All cattle were situated in individual rectangular pens with a dimension of 4 m (length) × 2 m (width) × 2 m (height) within an animal house (Figure 2), and were offered oaten chaff in buckets and water in automatic water bowls, ad libitum. The automated drinking bowls were approximately 75 cm above the floor on the left-hand side of the pens. Four commercial mineral block supplements (22 cm length × 22 cm width × 25 cm height) weighing approximately 16 kg, consisting of 7% urea and 10% molasses (Peak 50; Olsson’s Pacific Salt^®^, Yennora, NSW, Australia), were strapped to a metal frame (22.5 cm length × 22.5 cm width × 5 cm height) attached to the right-hand side of the pen’s panels and placed on the concrete floor alongside individual cattle.

After the seven-day habituation period, behaviours of the cattle were video-recorded for six hours daily (1000–1600 h) for seven days when access to mineral block supplements was provided. Video recordings were taken using four smartphone cameras [J5 Pro SM-J530Y (Samsung Engineering Co. Ltd., Gangdong-gu, Seoul, Korea), A9 (HTC Corp., Xindian, Taiwan, China), G6 Play (Motorola Inc., Chicago, IL, USA) and A5s (OPPO Mobile Telecommunications Corp., Ltd., Dongguan, Guangdong, China)]. The smartphones were placed on tripods and positioned 75 cm above the floor in front of the block supplements outside the pens. Video resolution for all phones was set at 1080 p (1920 × 1080 pixels) quality. Each smartphone was equipped with a 32 GB microSD card (SanDisk^®^, Milpitas, CA, USA) for video file storage. Timestamp Camera Free Application [30] was installed on the smartphones so that clock times on the display were automatically synchronised to AEDT Zone. Video files stored on the micro SD cards were then transferred daily onto a remote computer.

### 2.4. Video Analysis and Behaviour Classification

Each video file was observed and annotated using Sheep Movement Classification Interface software (version 1.1., UNE Precision Agriculture Research Group, Armidale, NSW, Australia) to generate annotated daily files for each steer in a .csv file format. Discrete events of individual behaviours were annotated to reflect the mutually exclusive behaviours of licking, eating, standing and lying (Table 1). The software time-stamped the beginning and the end of each event over a particular time regardless the type animals [6,10,31]. Each event was processed only if the cattle performed an observed behaviour for a minimum duration of 10 s to avoid multiple events merged in one epoch. To classify licking, all observed behaviours were partitioned into three groups of ethograms as follows:

1. Licking vs. eating vs. standing vs. lying.
2. Licking vs. eating vs. inactive (standing + lying).
3. Licking vs. non-licking (eating + standing + lying).

### 2.5. Processing of Raw Accelerometer Data

Accelerometer data were collected continuously for seven days and processed using the R statistics environment [32]. The average size of the .csv files (±SD) generated by each accelerometer throughout the study was 718 ± 17.5 MB. To facilitate analysis, these files were trimmed and extracted into separate files based on daily observational time (1000–1600 h) using the “lubridate” package [33]. Subsets of the accelerometer data were then annotated with corresponding behaviours. Time between accelerometers and clocks stamped on the video files had been automatically synchronised according to AEDT zone. All annotated files were then merged using the “dplyr” package [34] to create a new file for each deployed accelerometer.

### 2.6. Calculation of the Feature Relative Importance

The two datasets that contained *X*-, *Y*-, and *Z*-axes values and behaviour annotations were further discretised into a 10 s-time interval or windows size (epoch). Thus, there were 250 records required to create one row (or feature value) in each new dataset [11]. The 10-s time interval was chosen according to González et al. [35] who indicated that intervals longer than 10 s deteriorated the performance of behavioural classification model. Twenty movement features for each annotated behaviour were calculated, which included minimum (MIN_X,Y,Z_), maximum (MAX_X,Y,Z_), average (AVG_X,Y,Z_), and standard deviation (SD_X,Y,Z_) values of *X*-, *Y*-, and *Z*-axis, magnitude (MAG), movement variation (MVA), signal magnitude area (SMA), entropy (ENT), energy (ENG), pitch (PIT), roll (ROL) and inclination (INC). Mathematical formulas for these features are shown in Table 2 [6,8,11].

### 2.7. Development of Behaviour Classification Model

Each behaviour dataset for licking, eating, standing, and lying from all cattle was proportionally split into 70% (training) and 30% (testing) in the R statistic environment [11,36] using the “createDataPartition” function of the Classification and Regression Training (caret) package [37]. This process was independently performed in datasets from every deployment mode. The training dataset was assigned to develop the behaviour classification model, while the testing dataset was employed to validate performance of the model when applied to a different dataset [38]. In the model development, a 10-fold cross-validation was used to partition the training dataset into subsets of non-overlapping training and testing datasets for optimising parameter selection during the training process [23,39].

Four machine learning (ML) algorithms were employed to develop behaviour classification models: (1) decision trees (DT); (2) random forest (RF); (3) *k*-nearest neighbour (kNN); and (4) support vector machine (SVM). The DT algorithm constructs a group of binary trees based on the values of selected variables. It recursively splits the dataset into subsets with consistent values of the predictor variables [40]. The RF algorithm combines a set of decision trees with each tree having a random subset of variables that evenly distributed across the trees within the forest [41]. The kNN algorithm relies on the assumption that adjacent samples belong to a similar category [42]. The SVM algorithm establishes a hyperplane for splitting observations and maximising the distance of observations from the hyperplane. Hence, it is more appropriate for binary classification [43]. These algorithms were chosen as they are computationally easy to implement and have been used in previous studies [9,20,21,23,42,44,45].

### 2.8. Feature Selection

The “randomForest” and “varImpPlot” function of the “randomForest” package [46] were used on the training dataset to rank and visualise the most important features as prediction (dependent) variables according to their mean Gini values [6,39]. In “randomForest” setting, the number of variables that were arbitrarily sampled to split the junction of the tree (*mtry*) was set at 5 (approximately equal to square root of the number of prediction variables) and the number of trees (*ntree*) was set at 500. In RF and SVM algorithms, all features were used as prediction variables for developing the model while only the top three important features were selected for DT and kNN algorithms, respectively. Both DT and kNN are simple algorithms and only require a small number (3–5) of the top important features for model development based on their mean Gini values. Use of the top important features in the DT algorithm reduces the redundancies of the model development [47]. For the kNN, the higher number of features/variables used will lower the performance of the algorithm [48]. Previous studies on the accelerometer using DT and kNN have been described by Alvarenga et al. [49], Alvarenga et al. [11], and Shen et al. [42]. Analysis was performed using the “caret” package [37] within the R statistics environment.

### 2.9. Validation of Behaviour Classification Model

The behaviour classification models developed using the training dataset for each ML algorithm across the three ethograms in both deployment modes were independently applied to the testing dataset for validating their performance. The confusion matrix for each ML model prediction was computed using the “caret” package [37]. To determine the best model for each ethogram within the two accelerometer deployment modes, the overall accuracy, sensitivity, positive predictive value (PPV) and Cohen’s kappa coefficients of the predictions were then calculated based on the confusion matrix values using the following formulas:

(1) $$\mathrm{Overall}\;\mathrm{accuracy}=\frac{\left(\mathrm{TP}+\mathrm{TN}\right)}{\left(\mathrm{TP}+\mathrm{TN}+\mathrm{FP}+\mathrm{FN}\right)}$$

(2) $$\mathrm{Sensitivity}=\frac{\mathrm{TP}}{\left(\mathrm{TP}+\mathrm{FN}\right)}$$

(3) $$\mathrm{Positive}\;\mathrm{Predictive}\;\mathrm{Value}\;(\mathrm{PPV})=\frac{\mathrm{TP}}{\left(\mathrm{TP}+\mathrm{FP}\right)}$$

where TP (true positive) is the number of samples in which the observed behaviour was appropriately observed and classified, FP (false positive) is the number of samples in which other behaviours were classified as observed behaviour, TN (true negative) is the number of samples where other behaviours were appropriately observed and classified, and FN (false negative) is the number of samples in which the observed behaviour was classified as other behaviours [50]. Performance of the confusion matrix constituent was classified as: (1) high (90–100%), (4) moderate (80–89%), (5) low (70–79%), and (6) poor (<70%).

The inter-rated reliability test using Cohen’s kappa coefficients was likewise applied to select the best deployment for capturing values of the feature’s relative importance. The kappa statistic signifies the extent to which collection of the data represent the variables measured [51]. This would compare the accuracy of each accelerometer deployment in assessing features used to develop classification models. Kappa is suitable for imbalanced testing datasets without a very small minority class [52]. Under the definition of McHugh [51], the coefficient was classified as none (0–0.20), minimal (0.21–0.39), weak (0.40–0.59), moderate (0.60–0.79), strong (0.80–0.90), and almost perfect (>0.90).

## 3. Results

**Figure 3:**
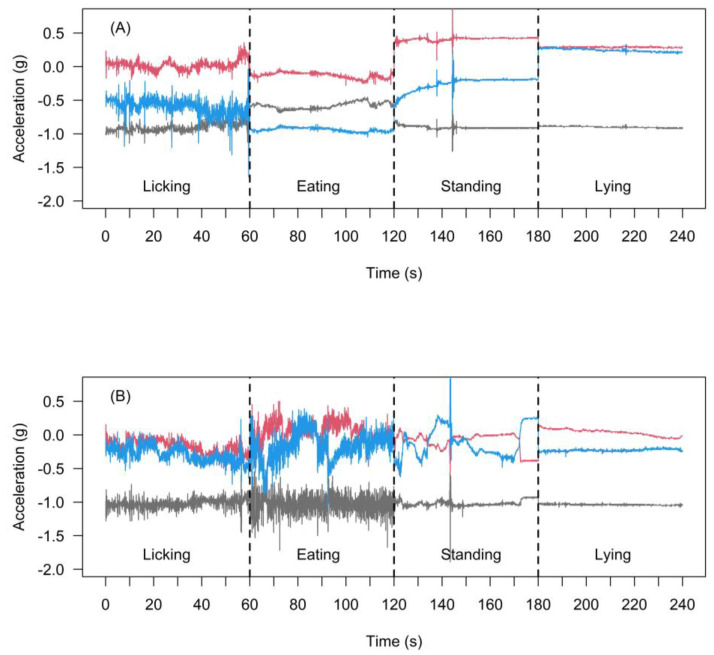
Raw values of the tri-axial accelerometer signals fitted on the neck-collar (**A**) and ear-tag (**B**) for licking, eating, standing, and lying behaviours at 25 Hz sampling rate over 60 s of observation. The grey, red, and blue lines represent *X-*, *Y-*, and *Z-* axes, respectively.

No aberrant behaviours resulting from accelerometer deployments were observed in any cattle throughout the experiment. The acceleration signals of the *X*-, *Y*-, and *Z*-axes sampled over a 60 s of observation from the neck-collar and ear-tag accelerometers for licking, eating, standing, and lying behaviours are depicted in Figure 3. Total number of samples (data points) obtained from the 10 s epoch for developing and validating behaviour classification models was 2362 for neck-collar and 2271 for ear-tag accelerometers, respectively. The proportion of samples across licking, eating, standing, and lying were consecutively 22.8% (*n* = 538), 26.2% (*n* = 618), 25.9% (*n* = 612,) and 25.1% (*n* = 594) for neck-collar and 26.2% (*n* = 594), 26.4% (*n* = 600), 25.5% (*n* = 580), and 21.9% (*n* = 497) for ear-tag deployment modes. The failure of the sensors to capture the acceleration signals has contributed to the unequal number of datapoints (samples) between neck-collar and ear-tag accelerometers.

### 3.1. Selection of the Most Important Features

**Figure 4:**
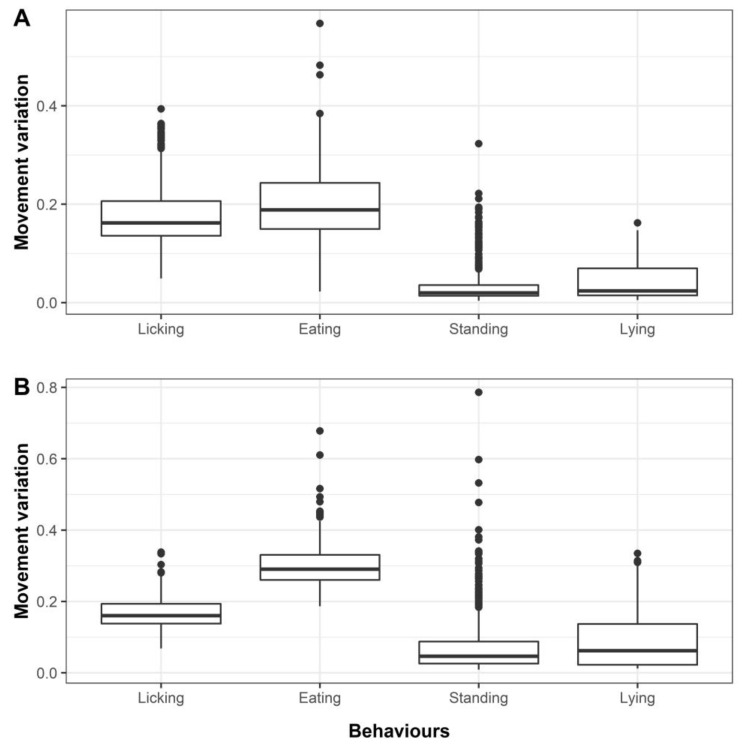
Distribution of movement variation (MVA) of the four mutually-exclusive behaviours within the neck-collar (**A**) and ear-tag accelerometers (**B**).

According to the mean Gini values, MVA and SDx were the first and second most important features for distinguishing licking from other observed behaviours across the three ethograms within both neck-collar and ear-tag deployment modes except for ethogram 3 of the neck-collar deployment (Table 3). The distribution of MVA of the four mutually exclusive behaviours for neck-collar and ear-tag accelerometers is displayed in Figure 3. The mean (±SD) of neck-collar and ear-tag MVA for eating behaviour was the highest among the four mutually-exclusive behaviours (0.20 (±0.07) and 0.30 (±0.05), respectively). Mean (±SD) MVA for licking was 0.18 (±0.06) and 0.17 (±0.04), lying 0.04 (±0.03) and 0.09 (±0.07), and standing 0.03 (±0.04) and 0.07 (±0.08) for the neck-collar and ear-tag, respectively. This sequential trend was consistent across both neck-collar and ear-tag accelerometer deployment modes (Figure 4A,B).

### 3.2. Overall Performance of the Behaviour Classification Models

The overall performance of ML algorithms in predicting behaviours of beef cattle using the testing dataset across the three different ethograms within the neck-collar and ear-tag accelerometers is presented in Table 4. The highest performance across all categories was consistently obtained from the RF algorithm with an accuracy ranging from moderate to high and kappa from strong to almost perfect (accuracy: 88–98%; kappa: 0.83–0.94) followed by SVM (accuracy: 83–97%; kappa: 0.56–0.93), kNN (accuracy: 71–94%; kappa: 0.61–0.89), and DT (accuracy: 65–91%; kappa: 0.52–0.77). The highest performance for the RF model was found in ethogram 3 of neck-collar accelerometer when differentiating licking and non-licking behaviours while the lowest performance of RF model was found in ethogram 1 of ear-tag accelerometer when classifying the four mutually-exclusive behaviours.

### 3.3. Performance of the Best Classification Model for Determination of Licking Behaviour

The performances of the RF algorithm model in classifying licking behaviour of beef cattle are described in Table 5 (ethogram 1), Table 6 (ethogram 2) and Table 7 (ethogram 3). Overall, the neck-collar deployed accelerometer had a slightly better performance based on the sensitivity and PPV than that of the ear-tag deployed accelerometer except for the PPV in ethogram 2 when distinguishing licking from eating and inactive behaviours. The RF model developed from neck-collar datasets achieved the highest sensitivity in ethogram 2 (99%) and the highest PPV in ethogram 3 (96%). When using ear-tag datasets, the uppermost sensitivity and PPV of the RF model were found in ethogram 1 (93%) and in ethogram 2 (95%), respectively.

## 4. Discussion

Dependency upon integration of radio frequency identification (RFID) and automatic feeding systems to remotely monitor supplement intake of beef cattle has prompted the use of more efficient and accurate technologies for the collection of individual information in larger herds without disrupting their daily routines and natural behaviours. Tri-axial accelerometers have the capability of accurately differentiating mutually-exclusive behaviours of grazing ruminants [6], and this is fundamental to predict individual feed intake based on time-spent feeding [53]. For cattle offered mineral block supplements, licking events and time spent licking have to be appropriately distinguished from other behaviours to develop an algorithm for predicting individual mineral block consumption. Supplementing cattle with mineral blocks is usually conducted while cattle are grazing in the paddock. This current study was designed as a pilot study to examine the capability of tri-axial accelerometers to differentiate the signals associated with licking and other behaviours. Therefore, only a small number of cattle were used and closely monitored while housed in pens. Further studies would need to be conducted with more animals to test the suitability of the sensor and algorithms under field conditions.

In this present study, MVA and SD_X_ were the top two features used to classify the licking behaviours of beef cattle by the ML algorithms employed on the tri-axial accelerometer data. This trend was consistent across five out of six ethograms (3 for each deployment mode). Gao et al. [8] explained that MVA is the variability of waveform length aggregate of amplitude, frequency and duration over the *X*-, *Y*- and *Z*-axes values while SD_X_ represents distribution of the signal within the *X*-axis values. Hence, the differentiation of *X*-axis values was evidence of apparent dorso-ventral moving direction recorded by neck-collar and ear-tag accelerometers when the event changed from licking to other behaviours. A recent study using an ear-tag accelerometer configured at 12.5 Hz with a 10 s time interval reported MVA and SD_X_ as the two most important features to classify grazing, lying, standing and walking events of sheep [39]. The presence of MVA and SD_X_ in our study indicated that the ML algorithms discriminated the behaviours based on the difference of movement patterns between behaviours.

For the neck-collar deployment, AVG_Z_ was the first important feature in ethogram 3 followed by SMA and MAX_Z_ and is the most consistent feature within the top three features in all ethograms. González et al. [35] found that SD of the vertical (up-down) acceleration from neck-collar accelerometer was more sensitive for differentiating grazing behaviours in cattle because of its ability to capture head positions. The change in *Z*-axis values in the present study signified that the neck-collar accelerometers captured the distinction of longitudinal (anterior-posterior) movements of the head when the cattle were licking. During licking the head is lowered and as the tongue protrudes, the head moves back and forth in the longitudinal plane. This might relate to the high accuracy of the neck-collar accelerometer in a situation where similar head orientation was captured from licking and biting behaviours. Also, SMA is a suitable measure to differentiate static and dynamic activities from the accelerometer signals [8,19,54]. Hence, the presence of SMA in ethogram 3 is indicative of the neck-collar accelerometer’s capability to distinguish between licking and inactive behaviours.

By using random forest ML algorithm, two deployment modes (neck-collar and ear-tag) of tri-axial accelerometers were capable of classifying licking by contrast with eating, standing, and lying behaviours with high accuracy (>90%; Table 4). The behaviour classification model for the RF algorithm was superior to that of SVM, kNN, and DT algorithms across all ethograms within the two deployment locations. Compared to other ML classifiers, RF has the capability to rank the most important predictor variables and to model multifarious interactions among variables to improve prediction accuracy [55]. Hence, instead of using all variables, RF randomly selects subsets of variables to determine the best split of each junction of the tree [43]. A study using a neck-collar accelerometer on dairy cows found that the RF algorithm was able of categorising grazing, ruminating, walking, and resting with an overall accuracy and kappa of 0.97 and 0.95, respectively [45]. The high accuracy of RF is mainly because of its robustness to noisy data and ability to handle non-linear correlated data [56].

The lower performance of the DT algorithm in this present study might be because of over-fitting the model and the hierarchical partitioning of each tree that reduces (1) the ability to categorise relationship between variables and (2) the effective sample sizes causing a difficulty in identifying rules and trends in each subsample [43]. It should be noted that in ethogram 2, inactive behaviour combined standing and lying while in ethogram 3, non-licking behaviour combined eating consisting of biting (head lowered) and chewing (head raised), standing (head raised) and lying (resting). Therefore, it was likely that the accelerometer signals from licking and biting when the cattle lowered the head would be misclassified, as the feeding bucket and mineral block supplement were positioned at a relatively similar height from the floor. This might be responsible for the moderate sensitivity of ear-tag deployment in ethogram 3 (<90%) and may have affected overall accuracy of the algorithm. In addition, lower PPV and sensitivity of the ear-tag accelerometer may have occurred because of a more flexible attachment of the sensor to the ear that increased the false positive rate. A lower ear-attached accelerometer (SensOor) performance was reported by Wolfger et al. [18], where negative predictive value and sensitivity of feeding class were 97% and 93%, respectively, with low specificity (70%) and poor PPV (54%). This was because of a high proportion of rumination that was categorised as feeding in their model.

In this current study, the behaviour classification model for the neck-collar tri-axial accelerometer was more accurate than the ear-tag tri-axial accelerometer, with Cohen’s Kappa coefficient for the neck-collar deployment model being also superior to the ear-tag deployment. The substantial agreement between actual and model-predicted behaviour was higher in the present study than studies with dairy cows by Bikker et al. [57] and dairy calves by Roland et al. [16] who found 0.77 and 0.68 of Cohen’s kappa value for eating and drinking using an ear-attached accelerometer. The lower kappa coefficient for the ear-tag accelerometer compared to that for the neck-collar was affected by complex and repetitive ear movements. Barwick et al. [6] reported that a possible interdependency of ear-tag acceleration signals from body movements might cause uniformity of the signals from different behaviours. Hence, rigid attachment of the sensors would maintain their orientation and consistent signal to generate accurate behaviour classification.

Apart from the lower performance of ear-tag based accelerometers compared to the neck-collar accelerometers, the practicalities of adoption in commercial contexts favour ear-attached sensors. The smaller size makes it less invasive to the cattle and costs less to implement per individual. Therefore, classification algorithms must be capable of dealing with interdependent dynamic accelerations. The potential of an ear-tag based sensor to accurately discriminate licking would be an improvement enabling measuring mineral block supplement intake based on time spent licking by individual cattle. It also offers versatility and is an efficient way to monitor and harness individual information particularly in an extensive environment. Advancements in remote monitoring systems using internet technology are required to remotely transmit the data from the ear-tag sensor to a central database system for improving production efficiency by reducing time of mustering for individual data collection. However, in commercial systems where cattle are already fitted with neck-collars for other purposes, measuring licking with neck-collar accelerometers would be ideal due to the greater accuracy with this deployment.

## 5. Conclusions

The behaviour classification model developed by random forest ML algorithm for both deployment modes performed well (accuracy: 88–98%; kappa: 0.83–0.94) compared to SVM, kNN and DT algorithms, with the neck-collar deployment mode performing slightly better in classifying licking behaviour within three different ethograms than the ear-tag deployment mode. This is partly because of the firm attachment of the sensor to the collar generating consistent orientation and acceleration signals. Movement of the ear independently from the body might also be responsible for lowering sensitivity and PPV of the model. For commercial use in large herds for grazing systems, however, the ear-tag deployment mode is more feasible and likely to be more cost efficient than the neck-collar deployment as current advancement in electronic ear tags for cattle allows attachment of automatic devices. This current study confirms that the accelerometer is a promising technology to differentiate between licking and other behaviours and provides important research evidence to continue applying this methodology to a paddock environment and to test the model performance in a commercial situation.

## Figures and Tables

**Table 1:** Behaviours description of individually confined cattle for ethogram classification.

Behaviour	Description
Licking	Minor limb movement in static standing position with head down approaching the mineral block supplement and the tongue presenting to the block surface.
Eating	Stationary with minor limb movements, head lowered approaching feeding bucket and biting the chaff or head raised with jaw movement (chewing or ruminating).
Standing	Standing stationary with head raised devoid of jaw movements.
Lying	Recumbent on the sternum or side with minor head movements and one side of the trunk was placed on the ground.

**Table 2:** Movement features calculated from tri-axial accelerometer *X-*, *Y-* and *Z-* axis values for each epoch.

Feature	Equation
Magnitude	$\frac{1}{n}\left(\overset{n}{\underset{i=1}{\sum }}\left(\sqrt{x_{i}^{2}+y_{i}^{2}+{\;z}_{i}^{2}}\right)\left(i\right)\right)$
Movement Variation	$\frac{1}{n-1}\left(\overset{n-1}{\underset{i=1}{\sum }}\left|x_{i+1}-{\;x}_{i}\right|\left(i\right)+\overset{n-1}{\underset{i=1}{\sum }}|y_{i+1}-{\;y}_{i}\left|\left(i\right)+\overset{n-1}{\underset{i=1}{\sum }}|z_{i+1}-{\;z}_{i}\right|\left(i\right)\right)$
Signal Magnitude Area	$\frac{1}{n}\left(\overset{n}{\underset{i=1}{\sum }}\left|x\left(i\right)\right|+\overset{n}{\underset{i=1}{\sum }}|y\left(i\right)\left|+\overset{n}{\underset{i=1}{\sum }}|z\left(i\right)\right|\right)$
Entropy	$\frac{1}{n}\left(\overset{n}{\underset{i=1}{\sum }}{\left(1+\left(x_{i}+{\;y}_{i}+{\;z}_{i}\right)\right)}^{2}*\;\ln{\left(1+\left(x_{i}+{\;y}_{i}+{\;z}_{i}\right)\right)}^{2}\left(i\right)\right)$
Energy	$\frac{1}{n}\left(\overset{n}{\underset{i=1}{\sum }}{\left(x_{i}^{2}+y_{i}^{2}+{\;z}_{i}^{2}\right)}^{2}\;\left(i\right)\right)$
Pitch	1n(∑i=1n(tan−1(−xi/(yi2+ zi2))∗ 180π)(i))
Roll	1n(∑i=1n(atan2(yi, zi)∗180π)(i))
Inclination	1n(∑i=1n( tan−1(xi2+ yi2zi)∗ 180π)(i))

**Table 3:** The mean Gini values of the three most important features across three different ethograms within two accelerometer deployment locations.

Ethogram	Neck-Collar		Ear-Tag	
	Feature	MGV	Feature	MGV
1	MVA	208	MVA	307
	SD_x_	120	SD_x_	124
	AVG_Z_	101	MIN_X_	74
2	MVA	218	MVA	298
	SD_x_	124	SD_x_	134
	AVG_Z_	96	ENG	78
3	AVG_z_	138	MVA	172
	SMA	97	SD_x_	57
	MAX_z_	59	AVG_z_	47

MGV = mean Gini value; MVA = movement variation; AVG = mean axis value, SD = standard deviation of axis; SMA = signal magnitude area; ENG = energy; MIN = minimum value of axis; MAX = maximum value of axis.

**Table 4:** Accuracy and kappa coefficient of machine learning (ML) predictions across three different ethograms within two accelerometer deployment modes. Bolded ML with asterisk symbol represents the highest prediction performance within each ethogram.

Deployment	Ethogram	ML	Accuracy (%)	Kappa
Neck-collar	1	DT	64.5	0.52
		RF *	**92.4**	**0.90**
		kNN	84.5	0.79
		SVM	87.6	0.83
	2	DT	85.3	0.77
		RF *	**94.8**	**0.92**
		kNN	92.8	0.89
		SVM	94.8	0.92
	3	DT	90.8	0.76
		RF *	**97.7**	**0.94**
		kNN	94.2	0.85
		SVM	97.2	0.93
Ear-tag	1	DT	68.8	0.58
		RF *	**87.5**	**0.83**
		kNN	70.7	0.61
		SVM	83.4	0.78
	2	DT	83.5	0.75
		RF *	**95.2**	**0.92**
		kNN	88.1	0.81
		SVM	93.1	0.89
	3	DT	90.3	0.75
		RF *	**95.7**	**0.89**
		kNN	91.2	0.77
		SVM	84.0	0.56

1 = licking vs. eating vs. standing vs. lying; 2 = licking vs. eating vs. inactive (standing + lying); 3 = licking vs. non-licking (eating + standing + lying); ML = machine learning; DT = decision trees; RF = random forest; kNN = k-nearest neighbour; SVM = support vector machine.

**Table 5:** Confusion matrix of the random forest algorithm in predicting four mutually-exclusive behaviours (ethogram 1) using testing datasets across two accelerometer deployment modes. Bold numbers represent correct prediction and italic numbers represent misclassification.

Deployment	Predicted Behaviour	Observed Behaviour ^1^				PPV (%)
		Licking	Eating	Standing	Lying	
Neck-collar	Licking	**183**	*8*	*2*	0	94.8
	Eating	*2*	**175**	*12*	*4*	90.7
	Standing	*1*	0	**154**	*14*	91.1
	Lying	0	*1*	*10*	**144**	92.9
**Sensitivity (%)**		98.4	95.1	86.5	88.9	
Ear-tag	Licking	**166**	*3*	*13*	*1*	90.7
	Eating	*7*	**173**	*8*	*1*	91.5
	Standing	*4*	*2*	**137**	29	79.7
	Lying	*1*	*1*	*15*	**119**	87.5
**Sensitivity (%)**		93.3	96.7	79.2	79.3	

^1^ = number of sample (data points) at 10 s epoch; PPV = positive predictive value.

**Table 6:** Confusion matrix of the random forest algorithm in predicting licking, eating, and inactive behaviours (ethogram 2) using testing datasets across two accelerometer deployment modes. Bold numbers represent correct prediction and italic numbers represent misclassification.

Deployment	Predicted Behaviour	Observed Behaviour ^1^			PPV (%)
		Licking	Eating	Inactive	
Neck-collar	Licking	**185**	*10*	*3*	93.4
	Eating	*1*	**169**	*18*	89.9
	Inactive	0	*5*	**317**	98.5
**Sensitivity (%)**		99.5	91.9	93.8	
Ear-tag	Licking	**165**	*5*	*4*	94.8
	Eating	*1*	**170**	*7*	95.5
	Inactive	*12*	*4*	**312**	95.1
**Sensitivity (%)**		92.7	95.0	96.6	

^1^ = number of sample (data points) at 10 s epoch; PPV = positive predictive value.

**Table 7:** Confusion matrix of the random forest algorithm in predicting licking and non-licking behaviours (ethogram 3) using testing datasets across two accelerometer deployment modes. Bold numbers represent correct prediction and italic number represents misclassification.

Deployment	Predicted Behaviour	Observed Behaviour ^1^		PPV (%)
		Licking	Non-Licking	
Neck-collar	Licking	**175**	*7*	96.2
	Non-licking	*9*	**515**	98.3
**Sensitivity (%)**		95.1	98.7	
Ear-tag	Licking	**160**	*11*	93.6
	Non-licking	*18*	**491**	96.5
**Sensitivity (%)**		89.9	97.8	

^1^ = number of sample (data points) at 10 s epoch; PPV = positive predictive value.

## Data Availability

The data presented in this study are available upon request from the corresponding author.
